# Characterization of warped product manifolds through the $W_{2}$-curvature tensor with applications to relativity

**DOI:** 10.1016/j.heliyon.2024.e36355

**Published:** 2024-08-22

**Authors:** Abdallah Abdelhameed Syied, Uday Chand De, Nasser Bin Turki, Gabriel-Eduard Vîlcu

**Affiliations:** a Department of Mathematics, Faculty of Science, Zagazig University, P.O. Box 44519, Zagazig, Egypt; b Department of Pure Mathematics, University of Calcutta, 35, Ballygaunge Circular Road Kolkata 700019, West Bengal, India; c Department of Mathematics, College of Science, King Saud University, P.O. Box 2455, Riyadh 11451, Saudi Arabia; d “Gheorghe Mihoc-Caius Iacob” Institute of Mathematical Statistics and Applied Mathematics of the Romanian Academy, 050711 Bucharest, Romania; e National University of Science and Technology Politehnica Bucharest, Faculty of Applied Sciences, Department of Mathematics and Informatics, 313 Splaiul Independenţei, 060042 Bucharest, Romania

**Keywords:** primary, 53C25, 53C50, secondary, 53C80, 53B20, Singly warped product manifolds, GRW space-times, $W_{2}$-curvature tensor, Einstein manifolds

## Abstract

We first investigate how the flatness and the symmetry of the $W_{2}$-tensor impact both the base manifold and the fiber manifold of a warped product manifold. In both cases, we determine the form of the $W_{2}$-tensor on both the fiber and the base manifolds. Additionally, we establish that the fiber manifold is of constant curvature, while the base manifold is Einstein. It is also proved that a $W_{2}$-curvature flat GRW space-time is perfect fluid and static. Furthermore, the space-time either represents dark matter era or the state equation has a specific form.

## 1 Introduction

The warped product manifold (WPM) $N=\overset{¯}{N}\times_{F}\overset{˜}{N}$ of two Riemannian manifolds $\left({\overset{¯}{N}}^{\overset{¯}{m}},\overset{¯}{g}\right)$ of dimension $\overset{¯}{m}$ and $\left({\overset{˜}{N}}^{\overset{˜}{m}},\overset{˜}{g}\right)$ of dimension $\overset{˜}{m}$ is the manifold $N=\overset{¯}{N}\times \overset{˜}{N}$ furnished with metric $g=\overset{¯}{g}+F\overset{˜}{g}$, where *F* denotes the warping function. The Riemannian manifold $\left({\overset{¯}{N}}^{\overset{¯}{m}},\overset{¯}{g}\right)$ is referred to as the base manifold while $\left(\overset{˜}{N},{\overset{˜}{g}}^{\overset{˜}{m}}\right)$ is named the fiber manifold. The function *F* is a positive $C^{\infty }$ function defined on the base manifold. WPMs are important mathematical objects that find applications in physics and mathematics. They provide a versatile framework for studying geometry, topology, gravity, cosmology, and other physical phenomena [1].

The warped product $\overset{¯}{N}\times_{F}\overset{˜}{N}$ is said to be generalized Robertson-Walker (GRW) space-time if its base manifold $\left(\overset{¯}{N},\overset{¯}{g}\right)$ is a 1-dimensional manifold with ${\overset{¯}{g}}_{11}=-1$ and its fiber manifold $\left(\overset{˜}{N},\overset{˜}{g}\right)$ is a (m−1)-Riemannian manifold [2], [3], [4]. In particular, when the fiber manifold has a constant curvature, the resulting warped product is referred to as a Robertson-Walker (RW) space-time [5], [6].

The curvature invariants hold significant importance in both differential geometry and general relativity. In differential geometry, such invariants serve as a scalar measure that quantifies the shape of a manifold, providing valuable information about its intrinsic and extrinsic geometry. This allows mathematicians to classify and study different types of manifolds, leading to a deeper understanding of their properties. In general relativity, the curvature invariants play a central role in Einstein's field equations, which relate the curvature of space-time to the distribution of matter and energy. By analyzing curvature invariants, physicists can unravel the intricate dynamics of gravitational forces, predict the behavior of particles and light in curved space-time, and explore the nature of the universe on both small and cosmological scales. In semi-Riemannian geometry, these invariants are scalar quantities constructed from various curvature tensors, the most famous being Riemann, Ricci, and conformal curvature tensors.

A significant curvature invariant which is named by $W_{2}$-tensor, was first coined by Pokhariyal and Mishra in 1970 and expressed as [7] (for more details about the curvature tensor see [8], [9]):

(1.1) $$W_{ijkl}=R_{ijkl}+\frac{1}{m-1}\left[g_{ik}R_{jl}-g_{jk}R_{il}\right],$$

where $R_{ijkl}$, $R_{ij=}g^{kl}R_{kijl}$, and $g_{ij}$ are the Riemann, the Ricci, the metric tensors respectively.

The $W_{2}$-tensor can be considered as an indicator of how a manifold deviates from having a constant curvature since a $W_{2}$-curvature flat manifold has a constant curvature. As a result, the significance of the $W_{2}$-tensor has been demonstrated by several authors in the study of certain structural aspects of differential geometry. For example, in [10], the $W_{2}$-tensor on a Kenmotsu manifolds was examined. Hui and Lemence investigated the $W_{2}$-tensor on generalized quasi-Einstein manifolds [11], the authors obtaining some geometrical information about such spaces when the manifold is $W_{2}$-flat or satisfies the condition $W_{2}\circ Ric=0$. Shenawy and Unal conducted a study of this curvature tensor on WPMs [12]. Moreover, Zengin [13] considered a space-time with $W_{2}$-tensor filled with a perfect fluid, showing that the energy-momentum tensor satisfying Einstein's equations with a cosmological constant is a quadratic conformal Killing tensor. Moreover, the author also obtains a necessary and sufficient condition for the energy momentum tensor to be a quadratic Killing tensor (see, e.g., [14] for the definition of (conformal) Killing tensors and examples). In [15], the authors provide a characterization of space-times admitting $W_{2}$-tensor in f(R,G)-gravity. Among various outcomes, they demonstrated that both the energy density and isotropic pressure remain constant. Additionally, they examined certain energy conditions.

This article is mainly organized to characterize the singly WPMs and the GRW space-times through the $W_{2}$-tensor. Firstly, we obtain characterizations of the singly WPMs through the flatness and the symmetry of the $W_{2}$-tensor. Secondly, we examine certain properties of the GRW space-times through the flatness of the $W_{2}$-tensor.

## 2 Preliminaries

The general structure of the metric $g_{ij}$, the Riemann curvature tensor $R_{ijkl}$, and the Ricci tensor $R_{ij}$ of $\overset{¯}{N}\times_{F}\overset{˜}{N}$ in terms of its factor Riemann manifolds $\left(\overset{¯}{N},\overset{¯}{g}\right)$, $\left(\overset{˜}{N},\overset{˜}{g}\right)$ and its warping function is well known (see [16], [17]). Locally, $g_{ij}$ is expressed as

(2.1) $$g_{ij}=\left\{ \begin{array}{cc} {\bar{g}}_{ab}\; & \text{for}\;i=a,\;j=b\\ F{\overset{˜}{g}}_{\alpha \beta }\; & \text{for}\;i=\alpha ,\;j=\beta .\\ 0\; & \text{otherwise} \end{array}\right.$$

The non-zero components of $R_{ijkl}$ are

(2.2) $$R_{abcd}={\overset{¯}{R}}_{abcd},$$

(2.3) $$R_{\alpha ab\beta }=\frac{-1}{2}T_{ab}{\overset{˜}{g}}_{\alpha \beta },$$

(2.4) $$R_{\alpha \beta \gamma \delta }=F{\overset{˜}{R}}_{\alpha \beta \gamma \delta }-\frac{1}{4}\overset{¯}{\Delta }F{\overset{˜}{G}}_{\alpha \beta \gamma \delta },$$

where ${\overset{˜}{G}}_{\alpha \beta \gamma \delta }={\overset{˜}{g}}_{\alpha \delta }{\overset{˜}{g}}_{\beta \gamma }-{\overset{˜}{g}}_{\alpha \gamma }{\overset{˜}{g}}_{\beta \delta }$, $\overset{¯}{\Delta }F={\bar{g}}^{ab}F_{a}F_{b}$, and $T_{ab}$ is a tensor of type (0,2) which satisfies

$$T_{ab}=F_{a,b}-\frac{1}{2F}F_{a}F_{b},\text{ and}\;T_{\alpha \beta }=T_{a\alpha }=0,$$

where “*comma”* refers to the covariant derivative.

Locally, $R_{ij}$ of $\overset{¯}{N}\times_{F}\overset{˜}{N}$ has the subsequent components

(2.5) $$R_{ab}={\overset{¯}{R}}_{ab}-\frac{\overset{˜}{m}}{2F}T_{ab},$$

(2.6) $$R_{\alpha \beta }={\overset{˜}{R}}_{\alpha \beta }-\frac{h}{2}{\overset{˜}{g}}_{\alpha \beta },$$

(2.7) $$R_{a\alpha }=0,$$

where $T={\overset{¯}{g}}^{ab}T_{ab}$ and $h=T+\frac{\overset{˜}{m}-1}{2F}\overset{¯}{\Delta }F$.

The Riemann curvature tensor's covariant derivative obeys

(2.8) $$R_{ab\alpha \beta ,c}=R_{ab\alpha \beta ,\gamma }=R_{\alpha \beta \gamma a,{}_{b}}=R_{\alpha ab\beta ,\gamma }=R_{abcd,\alpha }=R_{abc\alpha ,d}=0,$$

(2.9) $$R_{abcd,e}={\overset{¯}{R}}_{abcd,e},\;R_{\alpha \beta \gamma \delta ,\rho }=F{\overset{˜}{R}}_{\alpha \beta \gamma \delta ,\rho },\;R_{\alpha ac\beta ,b}=-\frac{{\overset{˜}{g}}_{\alpha \beta }}{2}T_{ac,b},$$

(2.10) $$R_{\alpha \beta \gamma \delta ,a}=-F_{a}{\overset{˜}{R}}_{\alpha \beta \gamma \delta }+\frac{1}{2}\left[\frac{F_{a}}{F}\overset{¯}{\Delta }F-\frac{1}{2}\partial_{a}\left(\overset{¯}{\Delta }F\right)\right]{\overset{˜}{G}}_{\alpha \beta \gamma \delta },$$

(2.11) $$R_{\alpha abc,\beta }=\frac{1}{2}\left[F_{e}{\overset{¯}{R}}_{abc}^{e}-\frac{1}{2F}\left(F_{b}T_{ab}-F_{c}T_{ab}\right)\right]{\overset{˜}{g}}_{\alpha \beta },$$

(2.12) $$R_{\alpha \beta \gamma a,\delta }=-\frac{1}{2}\left[F_{a}{\overset{˜}{R}}_{\alpha \beta \gamma \delta }+\frac{1}{2}\left(F_{e}T_{a}^{e}-\frac{1}{2}F_{a}\overset{¯}{\Delta }F\right){\overset{˜}{G}}_{\alpha \beta \gamma \delta }\right].$$

The component of the covariant derivative of $R_{ij}$ fulfills the subsequent equations

(2.13) $$R_{ab,c}={\overset{¯}{R}}_{ab,c}-\frac{\overset{˜}{m}}{2}{\left(\frac{T_{ab}}{F}\right)}_{,c},$$

(2.14) $$R_{\alpha \beta ,\gamma }={\overset{˜}{R}}_{\alpha \beta ,\gamma },\;\;\;\;R_{ab,\alpha }=R_{a\alpha ,b}=0,$$

(2.15) $$R_{\alpha \beta ,a}=\frac{-F_{a}}{F}{\overset{˜}{R}}_{\alpha \beta }+\frac{F_{a}}{2F}Q{\overset{˜}{g}}_{\alpha \beta }-\frac{1}{2}\left(\partial_{a}h\right){\overset{˜}{g}}_{\alpha \beta },$$

(2.16) $$R_{a\alpha ,\beta }=-\frac{F_{a}}{2F}\left[{\overset{˜}{R}}_{\alpha \beta }-\frac{Q}{2}{\overset{˜}{g}}_{\alpha \beta }\right]+\frac{1}{2}F_{e}\left[{\overset{¯}{R}}_{a}^{e}-\frac{\overset{˜}{m}}{2F}T_{a}^{e}\right]{\overset{˜}{g}}_{\alpha \beta },$$

where $Q=T+\frac{\overset{˜}{m}-1}{2F}\overset{¯}{\nabla }F$.

## 3 $W_{2}$-curvature flat WPMs

Here, our main objective is to examine the nature of the base and fiber manifolds of WPM $\overset{¯}{N}\times_{F}\overset{˜}{N}$ for the flatness of the $W_{2}$-tensor.

**The component**$W_{\alpha ab\beta }$**:** This component is
  (3.1) $$W_{\alpha ab\beta }=R_{\alpha ab\beta }+\frac{1}{m-1}\left[g_{\alpha b}R_{a\beta }-g_{ab}R_{\alpha \beta }\right].$$

The use of equations (2.1), (2.3), (2.6), and (2.7) in equation (3.1) implies

$$W_{\alpha ab\beta }=\frac{-1}{2}T_{ab}{\overset{˜}{g}}_{\alpha \beta }-\frac{1}{m-1}\left[{\overset{˜}{R}}_{\alpha \beta }-\frac{1}{2}h{\overset{˜}{g}}_{\alpha \beta }\right]{\overset{¯}{g}}_{ab}.$$

Assuming that the $W_{2}$-tensor is flat, it follows that

$${\overset{¯}{g}}_{ab}{\overset{˜}{R}}_{\alpha \beta }=\frac{-1}{2}\left(m-1\right)T_{ab}{\overset{˜}{g}}_{\alpha \beta }+\frac{1}{2}h{\overset{¯}{g}}_{ab}{\overset{˜}{g}}_{\alpha \beta }.$$

Multiplying by ${\overset{¯}{g}}^{ab}$, it is

(3.2) $${\overset{˜}{R}}_{\alpha \beta }=l{\overset{˜}{g}}_{\alpha \beta },$$

where $l=\frac{\left(1-m\right)}{2\overset{¯}{m}}T+\frac{1}{2}h$.

A simple contraction with ${\overset{˜}{g}}^{\alpha \beta }$ implies

(3.3) $$\overset{˜}{R}=\overset{˜}{m}l.$$

Utilizing equation (3.3) in equation (3.2), one reveals that

$${\overset{˜}{R}}_{\alpha \beta }=\frac{\overset{˜}{R}}{\overset{˜}{m}}{\overset{˜}{g}}_{\alpha \beta },$$

this demonstrates that the fiber manifold is nothing but an Einstein space.

Hence, we have

Theorem 1 *In a* $W_{2}$ *-curvature flat WPM, the fiber manifold becomes Einsteinian.*

**The component**$W_{\alpha \beta \gamma \delta }$**:** This is
  (3.4) $$W_{\alpha \beta \gamma \delta }=R_{\alpha \beta \gamma \delta }+\frac{1}{m-1}\left[g_{\alpha \gamma }R_{\beta \delta }-g_{\beta \gamma }R_{\alpha \delta }\right].$$

Utilizing equations (2.1), (2.6), and (2.4) in equation (3.4), we infer

(3.5) $$W_{\alpha \beta \gamma \delta }=F{\overset{˜}{R}}_{\alpha \beta \gamma \delta }+\frac{F}{m-1}\left[{\overset{˜}{g}}_{\alpha \gamma }{\overset{˜}{R}}_{\beta \delta }-{\overset{˜}{g}}_{\beta \gamma }{\overset{˜}{R}}_{\alpha \delta }\right]+S{\overset{˜}{G}}_{\alpha \beta \gamma \delta },$$

where $S=\frac{hF}{2\left(m-1\right)}-\frac{1}{4}\overset{¯}{\Delta }F$.

It may write in the subsequent structure

$$W_{\alpha \beta \gamma \delta }=F{\overset{˜}{W}}_{\alpha \beta \gamma \delta }-\frac{\overset{¯}{m}F}{\left(\overset{˜}{m}-1\right)\left(m-1\right)}\left[{\overset{˜}{g}}_{\alpha \gamma }{\overset{˜}{R}}_{\beta \delta }-{\overset{˜}{g}}_{\beta \gamma }{\overset{˜}{R}}_{\alpha \delta }\right]+S{\overset{˜}{G}}_{\alpha \beta \gamma \delta },$$

where,

$${\overset{˜}{W}}_{\alpha \beta \gamma \delta }={\overset{˜}{R}}_{\alpha \beta \gamma \delta }+\frac{1}{\overset{˜}{m}-1}\left[{\overset{˜}{g}}_{\alpha \gamma }{\overset{˜}{R}}_{\beta \delta }-{\overset{˜}{g}}_{\beta \gamma }{\overset{˜}{R}}_{\alpha \delta }\right].$$

Assuming that the $W_{2}$-tensor is flat, then it can be concluded that

(3.6) $${\overset{˜}{W}}_{\alpha \beta \gamma \delta }=\frac{\overset{¯}{m}}{\left(\overset{˜}{m}-1\right)\left(m-1\right)}\left[{\overset{˜}{g}}_{\alpha \gamma }{\overset{˜}{R}}_{\beta \delta }-{\overset{˜}{g}}_{\beta \gamma }{\overset{˜}{R}}_{\alpha \delta }\right]-\frac{S}{F}{\overset{˜}{G}}_{\alpha \beta \gamma \delta }.$$

Since the $W_{2}$-tensor is flat, it follows from equation (3.5) that

(3.7) $${\overset{˜}{R}}_{\alpha \beta \gamma \delta }=-\frac{1}{m-1}\left[{\overset{˜}{g}}_{\alpha \gamma }{\overset{˜}{R}}_{\beta \delta }-{\overset{˜}{g}}_{\beta \gamma }{\overset{˜}{R}}_{\alpha \delta }\right]-\frac{S}{F}{\overset{˜}{G}}_{\alpha \beta \gamma \delta }.$$

Contracting with ${\overset{˜}{g}}^{\alpha \delta }$, we get

$${\overset{˜}{R}}_{\beta \gamma }=\frac{\left(\overset{˜}{m}-1\right)}{m}\left[\frac{\overset{˜}{R}}{\left(\overset{˜}{m}-1\right)}-\frac{\left(m-1\right)S}{F}\right]{\overset{˜}{g}}_{\beta \gamma }.$$

Contracting with ${\overset{˜}{g}}^{\beta \gamma }$, we infer

(3.8) $$\overset{˜}{R}=-\frac{\overset{˜}{m}\left(\overset{˜}{m}-1\right)\left(m-1\right)}{\overset{¯}{m}F}S.$$

The last two equations imply

(3.9) $${\overset{˜}{R}}_{\beta \gamma }=\frac{\overset{˜}{R}}{\overset{˜}{m}}{\overset{˜}{g}}_{\beta \gamma }.$$

Using equations (3.8) and (3.9) in equation (3.7), the result is

$${\overset{˜}{R}}_{\alpha \beta \gamma \delta }=\frac{\overset{˜}{R}}{\overset{˜}{m}\left(\overset{˜}{m}-1\right)}{\overset{˜}{G}}_{\alpha \beta \gamma \delta }.$$

This implies that $\overset{˜}{N}$ is of constant curvature.

Hence, we can state

Theorem 2 *In a* $W_{2}$ *-curvature flat WPM,*

(1) *on the fiber manifold the* $W_{2}$ *-tensor can be expressed by equation* (3.6) *, and*
(2) *the fiber manifold* $\overset{˜}{N}$ *is Einstein.*

**The comonent**$W_{abcd}$**:** One may represent this component as follows:
  (3.10) $$W_{abcd}=R_{abcd}+\frac{1}{m-1}\left[g_{ac}R_{bd}-g_{bc}R_{ad}\right].$$

Utilizing equations (2.1), (2.2), and (2.5) in equation (3.10), we infer

(3.11) $$W_{abcd}={\overset{¯}{R}}_{abcd}+\frac{1}{\overset{¯}{m}-1}\left[{\overset{¯}{g}}_{ac}{\overset{¯}{R}}_{bd}-{\overset{¯}{g}}_{bc}{\overset{¯}{R}}_{ad}\right]-\frac{\overset{˜}{m}}{\left(\overset{¯}{m}-1\right)\left(m-1\right)}\left[{\overset{¯}{g}}_{ac}{\overset{¯}{R}}_{bd}-{\overset{¯}{g}}_{bc}{\overset{¯}{R}}_{ad}\right]+\frac{\overset{˜}{m}}{2\left(m-1\right)F}\left[{\overset{¯}{g}}_{bc}T_{ad}-{\overset{¯}{g}}_{ac}T_{bd}\right].$$

It may write as

$$W_{abcd}={\overset{¯}{W}}_{abcd}-\frac{\overset{˜}{m}}{\left(\overset{¯}{m}-1\right)\left(m-1\right)}\left[{\overset{¯}{g}}_{ac}{\overset{¯}{R}}_{bd}-{\overset{¯}{g}}_{bc}{\overset{¯}{R}}_{ad}\right]+\frac{\overset{˜}{m}}{2\left(m-1\right)F}\left[{\overset{¯}{g}}_{bc}T_{ad}-{\overset{¯}{g}}_{ac}T_{bd}\right],$$

where,

$${\overset{¯}{W}}_{abcd}={\overset{¯}{R}}_{abcd}+\frac{1}{\overset{¯}{m}-1}\left[{\overset{¯}{g}}_{ac}{\overset{¯}{R}}_{bd}-{\overset{¯}{g}}_{bc}{\overset{¯}{R}}_{ad}\right].$$

Assuming that the $W_{2}$-tensor is flat, we derive

(3.12) $${\overset{¯}{W}}_{abcd}=\frac{\overset{˜}{m}}{\left(\overset{¯}{m}-1\right)\left(m-1\right)}\left[{\overset{¯}{g}}_{ac}{\overset{¯}{R}}_{bd}-{\overset{¯}{g}}_{bc}{\overset{¯}{R}}_{ad}\right]-\frac{\overset{˜}{m}}{2\left(m-1\right)F}\left[{\overset{¯}{g}}_{bc}T_{ad}-{\overset{¯}{g}}_{ac}T_{bd}\right].$$

Since the $W_{2}$-tensor is flat, equation (3.11) implies

$${\overset{¯}{R}}_{abcd}=-\frac{1}{m-1}\left[{\overset{¯}{g}}_{ac}{\overset{¯}{R}}_{bd}-{\overset{¯}{g}}_{bc}{\overset{¯}{R}}_{ad}\right]-\frac{\overset{˜}{m}}{2\left(m-1\right)F}\left[{\overset{¯}{g}}_{bc}T_{ad}-{\overset{¯}{g}}_{ac}T_{bd}\right].$$

A contraction with ${\overset{¯}{g}}^{ad}$ gives

$${\overset{¯}{R}}_{bc}=-\frac{1}{m-1}\left[{\overset{¯}{R}}_{bc}-{\overset{¯}{g}}_{bc}\overset{¯}{R}\right]-\frac{\overset{˜}{m}}{2\left(m-1\right)F}\left[{\overset{¯}{g}}_{bc}T-T_{bc}\right].$$

$${\overset{¯}{R}}_{bc}=\frac{{\overset{¯}{g}}_{bc}}{m}\overset{¯}{R}-\frac{\overset{˜}{m}}{2mF}\left[{\overset{¯}{g}}_{bc}T-T_{bc}\right].$$

If $T_{bc}$ is proportional to ${\overset{¯}{g}}_{bc}$, then it is clear $\overset{¯}{N}$ is Einstein. Thus, we established the next result.

Theorem 3 *In a* $W_{2}$ *-curvature flat WPM,*

(1) *the* $W_{2}$ *-curvature on the base manifold is given by equation* (3.12) *, and*
(2) *the base manifold is Einstein if and only if* $T_{bc}$ *is proportional to the metric tensor.*

## 4 $W_{2}$-symmetric WPMs

The purpose of this section is to investigate the nature of the base and fiber manifolds of WPM $\overset{¯}{N}\times_{F}\overset{˜}{N}$ for the symmetry of the $W_{2}$-tensor.

Differentiating equation (1.1) covariantly, one finds

$$W_{ijkl,h}=R_{ijkl,h}+\frac{1}{m-1}\left[g_{ik}R_{jl,h}-g_{jk}R_{il,h}\right].$$

**The zero components:** The components that have a value of zero are the following:
  $$W_{ab\alpha \beta ,c}=W_{\alpha \beta \gamma a,b}=W_{abc\alpha ,d}=W_{ab\alpha \beta ,\gamma }=W_{abcd,\alpha }=0.$$
**The component**$\nabla_{\gamma }W_{\alpha ab\beta }$**:** This component is in the following form:
  $$W_{\alpha ab\beta ,\gamma }=R_{\alpha ab\beta ,\gamma }+\frac{1}{m-1}\left[g_{\alpha b}R_{a\beta ,\gamma }-g_{ab}R_{\alpha \beta ,\gamma }\right].$$

The previous equation turns into

$$W_{\alpha ab\beta ,\gamma }=\frac{-1}{m-1}{\overset{¯}{g}}_{ab}{\overset{˜}{R}}_{\alpha \beta ,\gamma },$$

where we have used equations (2.1), (2.8), (2.14), and (2.16).

Now, let's assume that the $W_{2}$-tensor exhibits symmetry. In this case,

$${\overset{˜}{R}}_{\alpha \beta ,\gamma }=0,$$

which demonstrates that $\overset{˜}{N}$ is Ricci symmetric [18].

Consequently, we can derive the following:

Theorem 4 *The fiber manifold of a* $W_{2}$ *-symmetric WPM is Ricci symmetric.*

**The component**$\nabla_{\rho }W_{\alpha \beta \gamma \delta }$**:** This component is described by the following expression:

$$W_{\alpha \beta \gamma \delta ,\rho }=R_{\alpha \beta \gamma \delta ,\rho }+\frac{1}{m-1}\left[g_{\alpha \gamma }R_{\beta \delta ,\rho }-g_{\beta \gamma }R_{\alpha \delta ,\rho }\right].$$

Making use of equations (2.1), (2.9), and (2.14) in the foregoing equation, we infer

(4.1) $$W_{\alpha \beta \gamma \delta ,\rho }=F{\overset{˜}{R}}_{\alpha \beta \gamma \delta ,\rho }+\frac{F}{m-1}\left[{\overset{˜}{g}}_{\alpha \gamma }{\overset{˜}{R}}_{\beta \delta ,\rho }-{\overset{˜}{g}}_{\beta \gamma }{\overset{˜}{R}}_{\alpha \delta ,\rho }\right].$$

One may write the foregoing equation as follows

(4.2) $$W_{\alpha \beta \gamma \delta ,\rho }=F{\overset{˜}{W}}_{\alpha \beta \gamma \delta ,\rho }-\frac{\overset{¯}{m}F}{\left(\overset{˜}{m}-1\right)\left(m-1\right)}\left[{\overset{˜}{g}}_{\alpha \gamma }{\overset{˜}{R}}_{\beta \delta ,\rho }-{\overset{˜}{g}}_{\beta \gamma }{\overset{˜}{R}}_{\alpha \delta ,\rho }\right],$$

where,

$${\overset{˜}{W}}_{\alpha \beta \gamma \delta }={\overset{˜}{R}}_{\alpha \beta \gamma \delta }+\frac{1}{\overset{˜}{m}-1}\left[{\overset{˜}{g}}_{\alpha \gamma }{\overset{˜}{R}}_{\beta \delta }-{\overset{˜}{g}}_{\beta \gamma }{\overset{˜}{R}}_{\alpha \delta }\right].$$

Considering that the $W_{2}$-tensor is symmetric, then equation (4.2) gives

$${\overset{˜}{W}}_{\alpha \beta \gamma \delta ,\rho }=\frac{\overset{¯}{m}}{\left(\overset{˜}{m}-1\right)\left(m-1\right)}\left[{\overset{˜}{g}}_{\alpha \gamma }{\overset{˜}{R}}_{\beta \delta ,\rho }-{\overset{˜}{g}}_{\beta \gamma }{\overset{˜}{R}}_{\alpha \delta ,\rho }\right],$$

which provides the expression for the covariant derivative of the $W_{2}$-tensor on the fiber manifold.

Based on equation (4.1), it can be inferred

$${\overset{˜}{R}}_{\alpha \beta \gamma \delta ,\rho }=\frac{-1}{m-1}\left[{\overset{˜}{g}}_{\alpha \gamma }{\overset{˜}{R}}_{\beta \delta ,\rho }-{\overset{˜}{g}}_{\beta \gamma }{\overset{˜}{R}}_{\alpha \delta ,\rho }\right].$$

Transvecting with ${\overset{˜}{g}}^{\alpha \delta }$, one gets

$${\overset{˜}{R}}_{\beta \gamma ,\rho }=\frac{{\overset{˜}{g}}_{\beta \gamma }}{m}{\overset{˜}{R}}_{,\rho }.$$

Contracting with ${\overset{˜}{g}}^{\beta \gamma }$, we find

$${\overset{˜}{R}}_{,\rho }=0.$$

Consequently,

$${\overset{˜}{R}}_{\beta \gamma ,\rho }=0.$$

It follows that

$${\overset{˜}{W}}_{\alpha \beta \gamma \delta ,\rho }=0,{\overset{˜}{R}}_{\alpha \beta \gamma \delta ,\rho }=0.$$

Consequently, we may present the subsequent theorem:

Theorem 5 *In a* $W_{2}$ *-symmetric WPM,*

(1) *the* $W_{2}$ *-tensor on* $\overset{˜}{N}$ *is symmetric, and*
(2) *the fiber manifold is a locally symmetric manifold.*

**The component**$\nabla_{b}W_{\alpha ac\beta }$**:** We can easily present this component as follows:
  $$W_{\alpha ac\beta ,b}=R_{\alpha ac\beta ,b}+\frac{1}{m-1}\left[g_{\alpha c}R_{a\beta ,b}-g_{ac}R_{\alpha \beta ,b}\right].$$

Utilizing equations (2.1), (2.9), (2.14), and (2.15) in the previous equation, one can be deduced

$$\left(m-1\right)W_{\alpha ac\beta ,b}=\frac{F_{b}}{F}{\overset{¯}{g}}_{ac}{\overset{˜}{R}}_{\alpha \beta }-\frac{F_{b}}{2F}Q{\overset{¯}{g}}_{ac}{\overset{˜}{g}}_{\alpha \beta }+\frac{1}{2}\left(\partial_{b}h\right){\overset{¯}{g}}_{ac}{\overset{˜}{g}}_{\alpha \beta }-\frac{\left(m-1\right)}{2}{\overset{˜}{g}}_{\alpha \beta }T_{ac,b}.$$

If the $W_{2}$-tensor is symmetric, then

$$\frac{F_{b}}{F}{\overset{¯}{g}}_{ac}{\overset{˜}{R}}_{\alpha \beta }+\frac{1}{2}\left(\partial_{b}h\right){\overset{¯}{g}}_{ac}{\overset{˜}{g}}_{\alpha \beta }=\frac{F_{b}}{2F}Q{\overset{¯}{g}}_{ac}{\overset{˜}{g}}_{\alpha \beta }+\frac{\left(m-1\right)}{2}{\overset{˜}{g}}_{\alpha \beta }T_{ac,b}$$

Contracting with ${\overset{¯}{g}}^{ac}$, one infers

$${\overset{˜}{R}}_{\alpha \beta }=\left[\frac{\left(m-1\right)F}{2\overset{¯}{m}F_{b}}T_{,b}+\frac{Q}{2}-\frac{F}{2F_{b}}\left(\partial_{b}h\right)\right]{\overset{˜}{g}}_{\alpha \beta }.$$

Multiplying with ${\overset{˜}{g}}^{\alpha \beta }$ infers

$$\overset{˜}{R}=\overset{˜}{m}\left[\frac{\left(m-1\right)F}{\overset{¯}{m}F_{b}}T_{,b}+\frac{Q}{2}-\frac{F}{2F_{b}}\left(\partial_{b}h\right)\right].$$

Merging the last two equation together, we easily get

$${\overset{˜}{R}}_{\alpha \beta }=\frac{\overset{˜}{R}}{\overset{˜}{m}}{\overset{˜}{g}}_{\alpha \beta }.$$

This implies that $\overset{˜}{N}$ is Einstein.

Thus, we have

Theorem 6 *The fiber of a* $W_{2}$ *-symmetric WPM is Einstein.*

**The component**$\nabla_{\beta }W_{\alpha abc}$**:** One can express this component as:
  (4.3) $$W_{\alpha abc,\beta }=R_{\alpha abc,\beta }+\frac{1}{m-1}\left[g_{\alpha b}R_{ac,\beta }-g_{ab}R_{\alpha c,\beta }\right].$$

With the help of equations (2.1), (2.11), (2.14), and (2.16), the equation (4.3) becomes

$$W_{\alpha abc,\beta }=\left[F_{e}{\overset{¯}{R}}_{abc}^{e}-\frac{1}{2F}\left(F_{b}T_{ac}-F_{c}T_{ab}\right)\right]\frac{{\overset{˜}{g}}_{\alpha \beta }}{2}-\frac{F_{e}}{2\left(m-1\right)}\left({\overset{¯}{R}}_{c}^{e}-\frac{\overset{˜}{m}}{2F}T_{c}^{e}\right){\overset{¯}{g}}_{ab}{\overset{˜}{g}}_{\alpha \beta }+\frac{F_{c}}{2\left(m-1\right)F}\left[{\overset{¯}{g}}_{ab}{\overset{˜}{R}}_{\alpha \beta }-\frac{Q}{2}{\overset{¯}{g}}_{ab}{\overset{˜}{g}}_{\alpha \beta }\right].$$

Assuming that the $W_{2}$-tensor is symmetric, then

$$\left[F_{e}{\overset{¯}{R}}_{abc}^{e}-\frac{1}{2F}\left(F_{b}T_{ac}-F_{c}T_{ab}\right)\right]\left(m-1\right){\overset{˜}{g}}_{\alpha \beta }=-\frac{F_{c}}{F}\left[{\overset{˜}{R}}_{\alpha \beta }-\frac{Q}{2}{\overset{˜}{g}}_{\alpha \beta }\right]{\overset{¯}{g}}_{ab}+F_{e}\left({\overset{¯}{R}}_{c}^{e}-\frac{\overset{˜}{m}}{2F}T_{c}^{e}\right){\overset{¯}{g}}_{ab}{\overset{˜}{g}}_{\alpha \beta }.$$

A contracting with ${\overset{¯}{g}}^{ab}$ gives

$${\overset{˜}{R}}_{\alpha \beta }=\left[\frac{FF_{e}}{F_{c}}\left({\overset{¯}{R}}_{c}^{e}-\frac{\overset{˜}{m}}{2F}T_{c}^{e}\right)+\frac{Q}{2}-\frac{\left(m-1\right)F}{\overset{¯}{m}F_{c}}\pi \right]{\overset{˜}{g}}_{\alpha \beta },$$

where $\pi =F_{e}{\overset{¯}{R}}_{c}^{e}-\frac{1}{2F}\left(F_{b}T_{c}^{b}-F_{c}T\right)$.

Contracting with $g^{\alpha \beta }$, we find

$$\overset{˜}{R}=\overset{˜}{m}\left[\frac{FF_{e}}{F_{c}}\left({\overset{¯}{R}}_{c}^{e}-\frac{\overset{˜}{m}}{2F}T_{c}^{e}\right)+\frac{Q}{2}-\frac{\left(m-1\right)F}{\overset{¯}{m}F_{c}}\pi \right].$$

Consequently,

$${\overset{˜}{R}}_{\alpha \beta }=\frac{\overset{˜}{R}}{\overset{˜}{m}}{\overset{˜}{g}}_{\alpha \beta },$$

this result being previously obtained.

**The component**$\nabla_{a}W_{\alpha \beta \gamma \delta }$**:** This component is
  (4.4) $$W_{\alpha \beta \gamma \delta ,a}=R_{\alpha \beta \gamma \delta ,a}+\frac{1}{m-1}\left[g_{\alpha \gamma }R_{\beta \delta ,a}-g_{\beta \gamma }R_{\alpha \delta ,a}\right].$$

Using equations (2.1), (2.10), and (2.15) in equation (4.4), we find

(4.5) $$W_{\alpha \beta \gamma \delta ,a}=-F_{a}{\overset{˜}{R}}_{\alpha \beta \gamma \delta }-\frac{F_{a}}{\left(m-1\right)}\left[{\overset{˜}{g}}_{\alpha \gamma }{\overset{˜}{R}}_{\delta \beta }-{\overset{˜}{g}}_{\beta \gamma }{\overset{˜}{R}}_{\alpha \delta }\right]+\frac{1}{2}\left[\frac{F_{a}}{F}\overset{¯}{\Delta }F-\frac{\partial_{a}\left(\overset{¯}{\Delta }F\right)}{2}+\frac{F\left(\partial_{a}h\right)}{2\left(m-1\right)}\right]{\overset{˜}{G}}_{\alpha \beta \gamma \delta }-\frac{QF_{a}}{2\left(m-1\right)}{\overset{˜}{G}}_{\alpha \beta \gamma \delta }.$$

It is intriguing to observe the following new form of the foregoing equation

$$W_{\alpha \beta \gamma \delta ,a}=-F_{a}{\overset{˜}{W}}_{\alpha \beta \gamma \delta }+\frac{\overset{¯}{m}F_{a}}{\left(m-1\right)\left(\overset{˜}{m}-1\right)}\left[{\overset{˜}{g}}_{\alpha \gamma }{\overset{˜}{R}}_{\beta \delta }-{\overset{˜}{g}}_{\beta \gamma }{\overset{˜}{R}}_{\alpha \delta }\right]+\frac{{\overset{˜}{G}}_{\alpha \beta \gamma \delta }}{2}\left[\frac{F_{a}}{F}\overset{¯}{\Delta }F-\frac{\partial_{a}\left(\overset{¯}{\Delta }F\right)}{2}+\frac{F\left(\partial_{a}h\right)}{2\left(m-1\right)}-\frac{QF_{a}}{\left(m-1\right)}\right],$$

where

$${\overset{˜}{W}}_{\alpha \beta \gamma \delta }={\overset{˜}{R}}_{\alpha \beta \gamma \delta }+\frac{1}{\overset{˜}{m}-1}\left[{\overset{˜}{g}}_{\alpha \gamma }{\overset{˜}{R}}_{\beta \delta }-{\overset{˜}{g}}_{\beta \gamma }{\overset{˜}{R}}_{\alpha \delta }\right].$$

Considering that the $W_{2}$-tensor is symmetric, we deduce

(4.6) $${\overset{˜}{W}}_{\alpha \beta \gamma \delta }=\frac{\overset{¯}{m}}{\left(m-1\right)\left(\overset{˜}{m}-1\right)}\left[{\overset{˜}{g}}_{\alpha \gamma }{\overset{˜}{R}}_{\beta \delta }-{\overset{˜}{g}}_{\beta \gamma }{\overset{˜}{R}}_{\alpha \delta }\right]+\left[\frac{\overset{¯}{\Delta }F}{F}-\frac{\partial_{a}\left(\overset{¯}{\Delta }F\right)}{2F_{a}}+\frac{F\left(\partial_{a}h\right)}{2\left(m-1\right)F_{a}}-\frac{Q}{\left(m-1\right)}\right]\frac{{\overset{˜}{G}}_{\alpha \beta \gamma \delta }}{2},$$

which gives the structure of the $W_{2}$-tensor on the fiber manifold.

It follows form equation (4.5) that

(4.7) $${\overset{˜}{R}}_{\alpha \beta \gamma \delta }=\left[\frac{\overset{¯}{\Delta }F}{F}-\frac{\partial_{a}\left(\overset{¯}{\Delta }F\right)}{2F_{a}}+\frac{F\left(\partial_{a}h\right)}{2\left(m-1\right)F_{a}}-\frac{Q}{\left(m-1\right)}\right]\frac{{\overset{˜}{G}}_{\alpha \beta \gamma \delta }}{2}-\frac{1}{\left(m-1\right)}\left[{\overset{˜}{g}}_{\alpha \gamma }{\overset{˜}{R}}_{\delta \beta }-{\overset{˜}{g}}_{\beta \gamma }{\overset{˜}{R}}_{\alpha \delta }\right].$$

Multiplying with ${\overset{˜}{g}}^{\alpha \delta }$, we obtain

$${\overset{˜}{R}}_{\beta \gamma }=\frac{\left(m-1\right)\left(\overset{˜}{m}-1\right)}{2m}\left[\frac{\overset{¯}{\Delta }F}{F}-\frac{\partial_{a}\left(\overset{¯}{\Delta }F\right)}{2F_{a}}+\frac{F\left(\partial_{a}h\right)}{2\left(m-1\right)F_{a}}-\frac{Q}{\left(m-1\right)}\right]{\overset{˜}{g}}_{\beta \gamma }+\frac{\overset{˜}{R}}{m}{\overset{˜}{g}}_{\beta \gamma }.$$

Transvecting with ${\overset{˜}{g}}^{\beta \gamma }$, we obtain

(4.8) $$\overset{˜}{R}=\frac{\overset{˜}{m}\left(m-1\right)\left(\overset{˜}{m}-1\right)}{2\overset{¯}{m}}\left[\frac{\overset{¯}{\Delta }F}{F}-\frac{\partial_{a}\left(\overset{¯}{\Delta }F\right)}{2F_{a}}+\frac{F\left(\partial_{a}h\right)}{2\left(m-1\right)F_{a}}-\frac{Q}{\left(m-1\right)}\right].$$

The combination of the last two equations leads to

(4.9) $${\overset{˜}{R}}_{\beta \gamma }=\frac{\overset{˜}{R}}{\overset{˜}{m}}{\overset{˜}{g}}_{\beta \gamma }.$$

This indicates that $\overset{˜}{N}$ is Einstein.

Using equations (4.8) and (4.9) in equation (4.7), one infers

$${\overset{˜}{R}}_{\alpha \beta \gamma \delta }=\frac{\overset{˜}{R}}{\overset{˜}{m}\left(\overset{˜}{m}-1\right)}\left[g_{\alpha \delta }g_{\beta \gamma }-g_{\alpha \gamma }g_{\beta \delta }\right],$$

which illustrates that $\overset{˜}{N}$ is of constant curvature.

Thus, the following theorem can be stated.

Theorem 7 *In a* $W_{2}$ *-symmetric WPM,*

(1) *the* $W_{2}$ *-tensor on the fiber manifold is given by equation* (4.6) *, and*
(2) *the fiber manifold is of constant curvature.*

**The component**$\nabla_{\delta }W_{\alpha \beta \gamma a}$**:** It is
  (4.10) $$W_{\alpha \beta \gamma a,\delta }=R_{\alpha \beta \gamma a,\delta }+\frac{1}{m-1}\left[g_{\alpha \gamma }R_{\beta a,\delta }-g_{\beta \gamma }R_{\alpha a,\delta }\right].$$

Utilizing equations (2.1), (2.12), and (2.16) in equation (4.10), we conclude

(4.11) $$W_{\alpha \beta \gamma a,\delta }=-\frac{1}{2}F_{a}{\overset{˜}{R}}_{\alpha \beta \gamma \delta }-\frac{F_{a}}{2\left(m-1\right)}\left[{\overset{˜}{g}}_{\alpha \gamma }{\overset{˜}{R}}_{\beta \delta }-{\overset{˜}{g}}_{\beta \gamma }{\overset{˜}{R}}_{\alpha \delta }\right]-\frac{{\overset{˜}{G}}_{\alpha \beta \gamma \delta }}{4\left(m-1\right)}\left[\left(m-1\right)\left(F_{e}T_{a}^{e}-\frac{1}{2}F_{a}\overset{¯}{\Delta }F\right)+QF_{a}\right]-\frac{FF_{e}}{2\left(m-1\right)}\left({\overset{¯}{R}}_{a}^{e}-\frac{\overset{˜}{m}}{2F}T_{a}^{e}\right){\overset{˜}{G}}_{\alpha \beta \gamma \delta }.$$

Equivalently, it is

$$W_{\alpha \beta \gamma a,\delta }=-\frac{1}{2}F_{a}{\overset{˜}{W}}_{\alpha \beta \gamma \delta }+\frac{\overset{¯}{m}F_{a}}{2\left(\overset{˜}{m}-1\right)\left(m-1\right)}\left[{\overset{˜}{g}}_{\alpha \gamma }{\overset{˜}{R}}_{\delta \beta }-{\overset{˜}{g}}_{\beta \gamma }{\overset{˜}{R}}_{\alpha \delta }\right]-\frac{{\overset{˜}{G}}_{\alpha \beta \gamma \delta }}{4\left(m-1\right)}\left[\left(m-1\right)\left(F_{e}T_{a}^{e}-\frac{1}{2}F_{a}\overset{¯}{\Delta }F\right)+QF_{a}\right]-\frac{FF_{e}}{2\left(m-1\right)}\left({\overset{¯}{R}}_{a}^{e}-\frac{\overset{˜}{m}}{2F}T_{a}^{e}\right){\overset{˜}{G}}_{\alpha \beta \gamma \delta },$$

where,

$${\overset{˜}{W}}_{\alpha \beta \gamma \delta }={\overset{˜}{R}}_{\alpha \beta \gamma \delta }+\frac{1}{\left(\overset{˜}{m}-1\right)}\left[{\overset{˜}{g}}_{\alpha \gamma }{\overset{˜}{R}}_{\beta \delta }-{\overset{˜}{g}}_{\beta \gamma }{\overset{˜}{R}}_{\alpha \delta }\right].$$

Let us assume that the $W_{2}$-tensor is symmetric, then

(4.12) $${\overset{˜}{W}}_{\alpha \beta \gamma \delta }=\frac{\overset{¯}{m}}{\left(\overset{˜}{m}-1\right)\left(m-1\right)}\left[{\overset{˜}{g}}_{\alpha \gamma }{\overset{˜}{R}}_{\delta \beta }-{\overset{˜}{g}}_{\beta \gamma }{\overset{˜}{R}}_{\alpha \delta }\right]-\frac{{\overset{˜}{G}}_{\alpha \beta \gamma \delta }}{2\left(m-1\right)F_{a}}\left[\left(m-1\right)\left(F_{e}T_{a}^{e}-\frac{1}{2}F_{a}\overset{¯}{\Delta }F\right)+QF_{a}\right]-\frac{FF_{e}}{\left(m-1\right)F_{a}}\left({\overset{¯}{R}}_{a}^{e}-\frac{\overset{˜}{m}}{2F}T_{a}^{e}\right){\overset{˜}{G}}_{\alpha \beta \gamma \delta },$$

which provides new expression for the $W_{2}$-tensor on the fiber manifold.

Since the $W_{2}$-tensor is symmetric, equation (4.11) becomes

(4.13) $${\overset{˜}{R}}_{\alpha \beta \gamma \delta }=-\frac{1}{m-1}\left[{\overset{˜}{g}}_{\alpha \gamma }{\overset{˜}{R}}_{\delta \beta }-{\overset{˜}{g}}_{\beta \gamma }{\overset{˜}{R}}_{\alpha \delta }\right]-\frac{1}{2F_{a}}\left(F_{e}T_{a}^{e}-\frac{1}{2}F_{a}\overset{¯}{\Delta }F\right){\overset{˜}{G}}_{\alpha \beta \gamma \delta }-\frac{{\overset{˜}{G}}_{\alpha \beta \gamma \delta }}{4\left(m-1\right)}\left[Q+\frac{2FF_{e}}{F_{a}}\left({\overset{¯}{R}}_{a}^{e}-\frac{\overset{˜}{m}}{2F}T_{a}^{e}\right)\right].$$

Multiplying with ${\overset{˜}{g}}^{\alpha \delta }$ infers

$${\overset{˜}{R}}_{\beta \gamma }=\left[\frac{\overset{˜}{R}}{m}-\frac{\left(m-1\right)\left(\overset{˜}{m}-1\right)}{2mF_{a}}\left(F_{e}T_{a}^{e}-\frac{1}{2}F_{a}\overset{¯}{\Delta }F\right)\right]{\overset{˜}{g}}_{\beta \gamma }-\frac{\left(\overset{˜}{m}-1\right)}{4m}\left[\left(T+\frac{\overset{˜}{m}-1}{2F}\overset{¯}{\nabla }F\right)+\frac{2FF_{e}}{F_{a}}\left({\overset{¯}{R}}_{a}^{e}-\frac{\overset{˜}{m}}{2F}T_{a}^{e}\right)\right]{\overset{˜}{g}}_{\beta \gamma }.$$

Transvecting with ${\overset{˜}{g}}^{\beta \gamma }$ provides

$$\overset{˜}{R}=-\frac{\overset{˜}{m}\left(\overset{˜}{m}-1\right)\left(m-1\right)}{2\overset{¯}{m}F_{a}}\left(F_{e}T_{a}^{e}-\frac{1}{2}F_{a}\overset{¯}{\Delta }F\right)-\frac{\overset{˜}{m}\left(\overset{˜}{m}-1\right)}{4\overset{¯}{m}}\left[Q+\frac{2FF_{e}}{F_{a}}\left({\overset{¯}{R}}_{a}^{e}-\frac{\overset{˜}{m}}{2F}T_{a}^{e}\right)\right].$$

The foregoing two equations give

$${\overset{˜}{R}}_{\beta \gamma }=\frac{\overset{˜}{R}}{\overset{˜}{m}}{\overset{˜}{g}}_{\beta \gamma }.$$

Utilizing the last two equations in equation (4.13), the result is

$${\overset{˜}{R}}_{\alpha \beta \gamma \delta }=\frac{\overset{˜}{R}}{\overset{˜}{m}\left(\overset{˜}{m}-1\right)}\left[{\overset{˜}{g}}_{\alpha \delta }{\overset{˜}{g}}_{\beta \gamma }-{\overset{˜}{g}}_{\alpha \gamma }{\overset{˜}{g}}_{\beta \delta }\right],$$

this result being mentioned in the previous theorem.

Based on equation (4.12), we can derive

Theorem 8 *In a* $W_{2}$ *-symmetric WPM, the* $W_{2}$ *-tensor on the fiber manifold can be given by equation* (4.12) *.*

**The component**$\nabla_{e}W_{abcd}$**:** This component can be written as follows:
  (4.14) $$W_{abcd,e}=R_{abcd,e}+\frac{1}{m-1}\left[g_{ac}R_{bd,e}-g_{bc}R_{ad,e}\right].$$

Using equations (2.1), (2.9), and (2.13) in equation (4.14) implies

(4.15) $$W_{abcd,e}={\overset{¯}{R}}_{abcd,e}+\frac{1}{m-1}\left[{\overset{¯}{g}}_{ac}{\overset{¯}{R}}_{bd,e}-{\overset{¯}{g}}_{bc}{\overset{¯}{R}}_{ad,e}\right]-\frac{\overset{˜}{m}}{2\left(m-1\right)}\left[{\overset{¯}{g}}_{ac}{\left(\frac{T_{bd}}{F}\right)}_{,e}-{\overset{¯}{g}}_{bc}{\left(\frac{T_{ad}}{F}\right)}_{,e}\right].$$

Also, it is

$$W_{abcd,e}={\overset{¯}{W}}_{abcd,e}-\frac{\overset{˜}{m}}{\left(\overset{¯}{m}-1\right)\left(m-1\right)}\left[{\overset{¯}{g}}_{ac}{\overset{¯}{R}}_{bd,e}-{\overset{¯}{g}}_{bc}{\overset{¯}{R}}_{ad,e}\right]-\frac{\overset{˜}{m}}{2\left(m-1\right)}\left[{\overset{¯}{g}}_{ac}{\left(\frac{T_{bd}}{F}\right)}_{,e}-{\overset{¯}{g}}_{bc}{\left(\frac{T_{ad}}{F}\right)}_{,e}\right],$$

where

$${\overset{¯}{W}}_{abcd}={\overset{¯}{R}}_{abcd}+\frac{1}{\overset{¯}{m}-1}\left[{\overset{¯}{g}}_{ac}{\overset{¯}{R}}_{bd}-{\overset{¯}{g}}_{bc}{\overset{¯}{R}}_{ad}\right].$$

If the $W_{2}$-tensor is symmetric, then

(4.16) $${\overset{¯}{W}}_{abcd,e}=\frac{\overset{˜}{m}}{\left(\overset{¯}{m}-1\right)\left(m-1\right)}\left[{\overset{¯}{g}}_{ac}{\overset{¯}{R}}_{bd_{,e}}-{\overset{¯}{g}}_{bc}{\overset{¯}{R}}_{ad,e}\right]+\frac{\overset{˜}{m}}{2\left(m-1\right)}\left[{\overset{¯}{g}}_{ac}{\left(\frac{T_{bd}}{F}\right)}_{,e}-{\overset{¯}{g}}_{bc}{\left(\frac{T_{ad}}{F}\right)}_{,e}\right],$$

and this gives the form of the covariant derivative of the $W_{2}$-tensor on the base manifold.

Equation (4.15) leads to

(4.17) $${\overset{¯}{R}}_{abcd,e}=-\frac{1}{m-1}\left[{\overset{¯}{g}}_{ac}{\overset{¯}{R}}_{bd,e}-{\overset{¯}{g}}_{bc}{\overset{¯}{R}}_{ad,e}\right]+\frac{\overset{˜}{m}}{2\left(m-1\right)}\left[{\overset{¯}{g}}_{ac}{\left(\frac{T_{bd}}{F}\right)}_{,e}-{\overset{¯}{g}}_{bc}{\left(\frac{T_{ad}}{F}\right)}_{,e}\right].$$

Contracting with ${\overset{¯}{g}}^{ad}$, one gets

(4.18) $${\overset{¯}{R}}_{bc,e}=\frac{{\overset{¯}{g}}_{bc}}{m}{\overset{¯}{R}}_{,e}+\frac{\overset{˜}{m}}{2m}\left[{\left(\frac{T_{bc}}{F}\right)}_{,e}-{\overset{¯}{g}}_{bc}{\left(\frac{T}{F}\right)}_{,e}\right].$$

A contraction with ${\overset{¯}{g}}^{bc}$ implies

(4.19) $${\overset{¯}{R}}_{,e}=\frac{\left(1-\overset{¯}{m}\right)}{2}{\left(\frac{T}{F}\right)}_{,e}.$$

Utilizing equation (4.19) in equation (4.18), we infer

(4.20) $${\overset{¯}{R}}_{bc,e}=\frac{1-m}{2m}{\overset{¯}{g}}_{bc}{\left(\frac{T}{F}\right)}_{,e}+\frac{\overset{˜}{m}}{2m}{\left(\frac{T_{bc}}{F}\right)}_{,e}.$$

Hence, we have

Theorem 9 *In a* $W_{2}$ *-symmetric WPM, the covariant derivative of the* $W_{2}$ *, the Riemann, and the Ricci tensors on* $\overset{¯}{N}$ *is given by equations* (4.16) *,* (4.17) *,* (4.20) *respectively.*

It is interesting to check that for $\frac{T_{bc}}{F}=constant$, one can get

$${\overset{¯}{R}}_{abcd,e}=0,{\overset{¯}{W}}_{abcd,e}=0,$$

which indicates that $\overset{¯}{N}$ is a locally symmetric and $W_{2}$-symmetric manifold.

Corollary 1 *The base manifold of a* $W_{2}$ *-symmetric WPM is a locally symmetric and* $W_{2}$ *-symmetric manifold, provided* $\frac{T_{bc}}{F}=constant$ *.*

## 5 Applications

This section provides characterizations of a GRW space-time through the flatness of the $W_{2}$-tensor. Note that in the following calculations we will take $F=\varphi^{2}$. Particularizing for GRW space-times, the metric tensor $g_{ij}$ satisfies

(5.1) $$g_{ij}=\left\{ \begin{array}{cc} {\bar{g}}_{tt}=-1\; & \text{for}\;i=t,\;j=t\\ \varphi^{2}{\overset{˜}{g}}_{\alpha \beta }\; & \text{for}\;i=\alpha ,\;j=\beta .\\ 0\; & \text{otherwise} \end{array}\right.$$

The non-zero local components of $R_{ijkl}$ are the following:

(5.2) $$R_{\alpha tt\beta }=-\overset{¨}{\varphi }\varphi {\overset{˜}{g}}_{\alpha \beta },$$

(5.3) $$R_{\alpha \beta \gamma \delta }=\varphi^{2}\left[{\overset{˜}{R}}_{\alpha \beta \gamma \delta }+{\overset{˙}{\varphi }}^{2}{\overset{˜}{G}}_{\alpha \beta \gamma \delta }\right].$$

The non-zero local components of $R_{ij}$ satisfy the following [19]:

(5.4) $$R_{tt}=-\frac{\left(m-1\right)\overset{¨}{\varphi }}{\varphi },R_{\alpha \beta }={\overset{˜}{R}}_{\alpha \beta }+\left(\overset{¨}{\varphi }\varphi +\left(m-2\right){\overset{˙}{\varphi }}^{2}\right){\overset{˜}{g}}_{\alpha \beta },$$

(5.5) $$R_{t\alpha }=0.$$

Let's begin by examining the first non-zero component of the $W_{2}$-tensor, namely

(5.6) $$W_{\alpha tt\beta }=R_{\alpha tt\beta }+\frac{1}{m-1}\left[g_{\alpha t}R_{t\beta }-g_{tt}R_{\alpha \beta }\right].$$

Making use of equations (5.1), (5.2), (5.4), and (5.5) in equation (5.6), one gets

$$W_{\alpha tt\beta }=\frac{1}{m-1}\left[{\overset{˜}{R}}_{\alpha \beta }+\left(m-2\right)\left({\overset{˙}{\varphi }}^{2}-\varphi \overset{¨}{\varphi }\right){\overset{˜}{g}}_{\alpha \beta }\right].$$

Assume that the $W_{2}$-tensor is flat, thus

(5.7) $${\overset{˜}{R}}_{\alpha \beta }=-\left(m-2\right)\left({\overset{˙}{\varphi }}^{2}-\varphi \overset{¨}{\varphi }\right){\overset{˜}{g}}_{\alpha \beta }.$$

A contraction of equation (5.7) with ${\overset{˜}{g}}^{{}_{\alpha \beta }}$ indicates

(5.8) $$\overset{˜}{R}=-\left(m-2\right)\left(m-1\right)\left({\overset{˙}{\varphi }}^{2}-\varphi \overset{¨}{\varphi }\right).$$

The last two equations implies

$${\overset{˜}{R}}_{\alpha \beta }=\frac{\overset{˜}{R}}{m-1}{\overset{˜}{g}}_{\alpha \beta },$$

which illustrates that $\overset{˜}{N}$ is Einstein.

As the fiber manifold $\overset{˜}{N}$ is Einstein, $\overset{˜}{R}$ is constant. Consequently,

$${\overset{˙}{\varphi }}^{2}-\varphi \overset{¨}{\varphi }=constant.$$

The component $W_{\alpha \beta \gamma \delta }$ is

$$W_{\alpha \beta \gamma \delta }=R_{\alpha \beta \gamma \delta }+\frac{1}{m-1}\left[g_{\alpha \gamma }R_{\beta \delta }-g_{\beta \gamma }R_{\alpha \delta }\right].$$

It follows from equations (5.1), (5.3), and (5.4) that

(5.9) $$W_{\alpha \beta \gamma \delta }=\varphi^{2}\{{\overset{˜}{R}}_{\alpha \beta \gamma \delta }+\frac{1}{\left(m-2\right)}({\overset{˜}{g}}_{\alpha \gamma }{\overset{˜}{R}}_{\beta \delta }-{\overset{˜}{g}}_{\beta \gamma }{\overset{˜}{R}}_{\alpha \delta })\}-\frac{\varphi^{2}}{\left(m-1\right)\left(m-2\right)}[{\overset{˜}{g}}_{\alpha \gamma }{\overset{˜}{R}}_{\beta \delta }-{\overset{˜}{g}}_{\beta \gamma }{\overset{˜}{R}}_{\alpha \delta }]+\frac{\varphi^{2}}{\left(m-1\right)}\left({\overset{˙}{\varphi }}^{2}-\varphi \overset{¨}{\varphi }\right){\overset{˜}{G}}_{\alpha \beta \gamma \delta }.$$

This equation can be rewritten as follows

$$W_{\alpha \beta \gamma \delta }=\varphi^{2}{\overset{˜}{W}}_{\alpha \beta \gamma \delta }-\frac{\varphi^{2}}{\left(m-1\right)\left(m-2\right)}\left[{\overset{˜}{g}}_{\alpha \gamma }{\overset{˜}{R}}_{\beta \delta }-{\overset{˜}{g}}_{\beta \gamma }{\overset{˜}{R}}_{\alpha \delta }\right]+\frac{\varphi^{2}}{m-1}\left({\overset{˙}{\varphi }}^{2}-\varphi \overset{¨}{\varphi }\right){\overset{˜}{G}}_{\alpha \beta \gamma \delta },$$

where

$${\overset{˜}{W}}_{\alpha \beta \gamma \delta }={\overset{˜}{R}}_{\alpha \beta \gamma \delta }+\frac{1}{\left(m-2\right)}[{\overset{˜}{g}}_{\alpha \gamma }{\overset{˜}{R}}_{\beta \delta }-{\overset{˜}{g}}_{\beta \gamma }{\overset{˜}{R}}_{\alpha \delta }].$$

Assuming that $W_{2}$-tensor is flat, then it follows that

$${\overset{˜}{W}}_{\alpha \beta \gamma \delta }=\frac{1}{\left(m-1\right)\left(m-2\right)}\left[{\overset{˜}{g}}_{\alpha \gamma }{\overset{˜}{R}}_{\beta \delta }-{\overset{˜}{g}}_{\beta \gamma }{\overset{˜}{R}}_{\alpha \delta }\right]-\frac{1}{m-1}\left({\overset{˙}{\varphi }}^{2}-\varphi \overset{¨}{\varphi }\right){\overset{˜}{G}}_{\alpha \beta \gamma \delta }.$$

Equation (5.9) leads to

(5.10) $${\overset{˜}{R}}_{\alpha \beta \gamma \delta }=\frac{-1}{\left(m-1\right)}\left[{\overset{˜}{g}}_{\alpha \gamma }{\overset{˜}{R}}_{\beta \delta }-{\overset{˜}{g}}_{\beta \gamma }{\overset{˜}{R}}_{\alpha \delta }\right]-\frac{1}{m-1}\left({\overset{˙}{\varphi }}^{2}-\varphi \overset{¨}{\varphi }\right){\overset{˜}{G}}_{\alpha \beta \gamma \delta }.$$

Substituting form equation (5.7) in equation (5.10), one infers

$${\overset{˜}{R}}_{\alpha \beta \gamma \delta }=-\left({\overset{˙}{\varphi }}^{2}-\varphi \overset{¨}{\varphi }\right){\overset{˜}{G}}_{\alpha \beta \gamma \delta }.$$

From equation (5.8) in last equation, we see

$${\overset{˜}{R}}_{\alpha \beta \gamma \delta }=\frac{\overset{˜}{R}}{\left(m-1\right)\left(m-2\right)}{\overset{˜}{G}}_{\alpha \beta \gamma \delta },$$

this means that the fiber manifold $\overset{˜}{N}$ is of constant curvature.

Thus, we get

Theorem 10 *A* $W_{2}$ *-curvature flat GRW space-time has the following properties:*

(1) *The* $W_{2}$ *-tensor on the fiber manifold* $\overset{˜}{N}$ *is expressed as*
  $${\overset{˜}{W}}_{\alpha \beta \gamma \delta }=\frac{1}{\left(m-1\right)\left(m-2\right)}\left[{\overset{˜}{g}}_{\alpha \gamma }{\overset{˜}{R}}_{\beta \delta }-{\overset{˜}{g}}_{\beta \gamma }{\overset{˜}{R}}_{\alpha \delta }\right]-\frac{1}{m-1}\left({\overset{˙}{\varphi }}^{2}-\varphi \overset{¨}{\varphi }\right){\overset{˜}{G}}_{\alpha \beta \gamma \delta }.$$
(2) *The fiber manifold* $\overset{˜}{N}$ *is of constant curvature, and*
(3) *The warping function satisfies* ${\overset{˙}{\varphi }}^{2}-\varphi \overset{¨}{\varphi }=constant$ *.*

In [19], Sanchez proved that a GRW space-time *N* becomes perfect fluid if and only if the fiber manifold $\overset{˜}{N}$ is Einstein.

Therefore, we can derive

Corollary 2 *A* $W_{2}$ *-curvature flat GRW space-time is a perfect fluid space-time.*

The Weyl tensor is [20], [21]

$${\text{C}}_{ijkl}={\text{R}}_{ijkl}-\frac{1}{m-2}\{g_{il}R_{jk}+g_{jk}R_{il}-g_{ik}R_{jl}-g_{jl}R_{ik}\}+\frac{R}{\left(m-1\right)\left(m-2\right)}\{g_{il}g_{jk}-g_{ik}g_{jl}\},$$

and it divergence is in the following form [22], [23]

$$\nabla_{h}C_{ijk}^{h}=\frac{m-3}{m-2}\left[\left(\nabla_{k}R_{ij}-\nabla_{j}R_{ik}\right)-\frac{1}{2\left(m-1\right)}\left(g_{ij}\nabla_{k}R-g_{ik}\nabla_{j}R\right)\right].$$

A Weyl curvature tensor is called harmonic if its divergence vanishes, that is,

$$\nabla_{h}C_{ijk}^{h}=0.$$

The conjunction of two findings presented in [19], [24] leads to the implication that a perfect fluid GRW space-time can be characterized as Weyl harmonic, that is, $\nabla_{h}C_{ijk}^{h}=0$.

Corollary 3 *A* $W_{2}$ *-curvature flat GRW space-time is Weyl harmonic.*

It has been demonstrated that a 4-dimensional GRW space-time satisfying the condition $\nabla_{h}C_{ijk}^{h}=0$ is a RW space-time [25]. Hence, we have the next result.

Corollary 4 *A* 4*-dimensional* $W_{2}$*-curvature flat GRW space-time is a Robertson-Walker space-time.*

On a GRW space-time Mantica and Molinari proved that the following two conditions are equivalent:

$$\nabla_{h}C_{ijk}^{h}=0⟺u^{h}C_{ijkh}=0.$$

In a perfect fluid space-time, the Ricci tensor takes the following expression [25]

(5.11) $$R_{ij}=au_{i}u_{j}+bg_{ij}-(m-2)u^{l}u^{k}C_{lijk},$$

where $a=\frac{R-m\zeta }{m-1}$, $b=\frac{R-\zeta }{m-1}$.

Differentiating the previous equation covariantly, we deduce

$$\nabla_{n}R_{ij}=u_{i}u_{j}a_{n}+a\left[u_{j}\left(\nabla_{n}u_{i}\right)+u_{i}\left(\nabla_{n}u_{j}\right)\right]+g_{ij}b_{n}-(m-2)\left[\left(\nabla_{n}u^{l}\right)u^{k}+\left(\nabla_{n}u^{k}\right)u^{l}\right]C_{lijk}-(m-2)u^{l}u^{k}\left(\nabla_{n}C_{lijk}\right),$$

where $a_{n}=\nabla_{n}a$ and $b_{n}=\nabla_{n}b$.

Since a $W_{2}$-curvature flat GRW space-time is of constant curvature, it follows that $\nabla_{n}R_{ijkl}=0$, $\nabla_{n}R_{ij}=0$ and consequently $\nabla_{n}C_{ijkl}=0$. As a result, the previous equation becomes

$$u_{i}u_{j}a_{n}+a\left[u_{j}\left(\nabla_{n}u_{i}\right)+u_{i}\left(\nabla_{n}u_{j}\right)\right]+g_{ij}b_{n}-(m-2)\left[\left(\nabla_{n}u^{l}\right)u^{k}C_{lijk}+\left(\nabla_{n}u^{k}\right)u^{l}\right]C_{lijk}=0.$$

Since $\nabla_{h}C_{ijk}^{h}=0$, it implies that $u^{h}C_{ijkh}=0$. The foregoing equation becomes

(5.12) $$u_{i}u_{j}a_{n}+a\left[u_{j}\left(\nabla_{n}u_{i}\right)+u_{i}\left(\nabla_{n}u_{j}\right)\right]+g_{ij}b_{n}=0.$$

Contracting of equation (5.11), we find

R=−a+mb.

Applying the operator $\nabla_{m}$ of the both sides, we deduce

$$\nabla_{n}R=-\nabla_{n}a+m\nabla_{n}b.$$

As mentioned above R is constant, thus

(5.13) $$a_{n}=mb_{n}.$$

Making use of equation (5.13) in equation (5.12), we reveal that

$$a\left[u_{j}\left(\nabla_{n}u_{i}\right)+u_{i}\left(\nabla_{n}u_{j}\right)\right]+\left[g_{ij}+mu_{i}u_{j}\right]b_{n}=0.$$

Transvecting with $u^{i}$, it can be deduced that

$$a\nabla_{n}u_{j}=\left[1-m\right]u_{j}b_{n}.$$

A contraction with $u^{j}$ implies

$$b_{n}=0.$$

Consequently,

(5.14) $$\nabla_{n}u_{j}=0,$$

which means that the vector field $u_{j}$ is parallel.

Contracting the aforementioned equation with $u^{j}$, we conclude that

(5.15) $${\overset{˙}{u}}_{n}=u^{j}\nabla_{n}u_{j}=0,$$

which means that the time-like unit vector field $u_{n}$ is acceleration-free [26].

Equations (5.14) and (5.15) demonstrate that $u_{j}$ is vorticity-free:

$$\mu_{ij}=\frac{1}{2}\left(\nabla_{i}u_{j}-\nabla_{j}u_{i}\right)+\frac{1}{2}{\overset{˙}{u}}_{j}u_{i}+u_{j}{\overset{˙}{u}}_{i}=0.$$

In a space-time *N*, $\nabla_{i}u_{j}$ has standard decomposition with geometric meaning [27]:

$$\nabla_{i}u_{j}=\frac{\nabla_{k}u^{k}}{m-1}\left(u_{i}u_{j}+g_{ij}\right)-u_{i}{\overset{˙}{u}}_{j}+\mu_{ij}+\sigma_{ij},$$

being $\sigma_{ij}$ the shear tensor which is traceless and symmetric.

It is clearly visible that

$$\sigma_{ij}=0,$$

which means that the shear tensor vanishes.

Thus, we have

Theorem 11 *In a* $W_{2}$ *-curvature flat GRW space-time, the velocity vector field* $u_{j}$ *is acceleration-free, vorticity-free, and shear-free.*

A space-time *N* is considered static if it possesses a unit vector field $u_{i}$ that is time-like and satisfies the following two conditions [23], [28], [29]:

$$\nabla_{i}u_{j}=-u_{i}{\overset{˙}{u}}_{j},u_{j}{\overset{¨}{u}}_{i}-u_{i}{\overset{¨}{u}}_{j}=0.$$

Equations (5.14) and (5.15) show that the previous two conditions are satisfied.

Theorem 12 *A* $W_{2}$ *-curvature flat GRW space-time is static.*

The Ricci tensor for a perfect fluid space-time gains the form [30], [31]

(5.16) $$R_{ij}=\kappa \left(\mu +p\right)u_{i}u_{j}+\frac{\kappa }{\left(2-m\right)}\left(p-\mu \right)g_{ij},$$

where *κ*, *μ*, and *p* refers to the gravitational constant, the energy density, and the isotropic pressure, respectively.

The previous equation leads to

$$\left(\nabla_{h}\nabla_{k}-\nabla_{k}\nabla_{h}\right)R_{ij}=\left(\mu +p\right)u_{i}\left(\nabla_{h}\nabla_{k}-\nabla_{k}\nabla_{h}\right)u_{j}+\left(\mu +p\right)u_{j}\left(\nabla_{h}\nabla_{k}-\nabla_{k}\nabla_{h}\right)u_{i}.$$

The Ricci tensor is semi-symmetric, that is,

$$\left(\nabla_{h}\nabla_{k}-\nabla_{k}\nabla_{h}\right)R_{ij}=0.$$

Therefore,

$$\left(\mu +p\right)u_{i}\left(\nabla_{h}\nabla_{k}-\nabla_{k}\nabla_{h}\right)u_{j}+\left(\mu +p\right)u_{j}\left(\nabla_{h}\nabla_{k}-\nabla_{k}\nabla_{h}\right)u_{i}=0.$$

Multiplying by $u^{i}$, we find

$$-\left(\mu +p\right)\left(\nabla_{h}\nabla_{k}-\nabla_{k}\nabla_{h}\right)u_{j}+\left(\mu +p\right)u_{j}u^{i}\left(\nabla_{h}\nabla_{k}-\nabla_{k}\nabla_{h}\right)u_{i}=0.$$

It is well-know that

$$u^{i}\left(\nabla_{h}\nabla_{k}-\nabla_{k}\nabla_{h}\right)u_{i}=0,$$

and thus

$$\left(\mu +p\right)\left(\nabla_{h}\nabla_{k}-\nabla_{k}\nabla_{h}\right)u_{j}=0,\left(\mu +p\right)R_{hkj}^{\;\;\;m}u_{m}=0.$$

This observation leads us to consider the following two potential outcomes:

(1) If $R_{hkj}^{\;\;\;m}u_{m}\neq 0$, it implies that μ+p=0, indicating that the space-time represents dark matter era [32], [29].
(2) If μ+p≠0, then it follows that $R_{hkj}^{\;\;\;m}u_{m}=0$. By performing a straightforward contraction with $g^{hj}$, we obtain $R_{mk}u^{m}=0$. Transvecting equation (5.16) with $u^{i}$, with the aid of this result one finds
  (5.17) $$\mu =-\frac{\left(m-3\right)}{m-1}p.$$
  From the above discussion, we have the following:

Theorem 13 *A* $W_{2}$ *-curvature flat GRW space-time either represents dark matter era or the state equation is given by equation* (5.17) *.*

For m=4, equation (5.17) gives $\frac{p}{\mu }=-3$. This means that the space-time denotes phantom era [33].

Corollary 5 *A* 4*-dimensional* $W_{2}$*-curvature flat GRW space-time either represents dark matter era or phantom era.*

## 6 Examples

Example 1 The Lorentz-Minkowski space-time $R_{1}^{m+1}$ provides us the simplest example to illustrate Theorem 1, Theorem 2. Recall that $R_{1}^{m+1}$ is a trivial warped product having the base R, fiber $R^{m}$ (equipped with the Euclidean metric of constant sectional curvature 0) and warping function F=1. In this case, it is clear that $R_{1}^{m+1}$ is $W_{2}$-curvature flat due to the fact that it is flat (see [19]). Moreover, the fiber manifold is clearly Einstein.

Example 2 A non-trivial example of $W_{2}$-curvature flat manifold to illustrate Theorem 1, Theorem 2 can be constructed in the following way. Suppose $N=I\times_{F}\overset{˜}{N}$ is the warped product of an open and connected interval *I* of R (endowed with the Euclidean metric tensor on *I*) with a 3-dimensional Riemannian manifold $\left(\overset{˜}{N},\overset{˜}{g}\right)$ of constant sectional curvature *κ*. Then, applying Theorem 9 from [19], it follows that *N* is a $W_{2}$-curvature flat manifold if the warping function *F* is chosen such that ${\overset{˙}{F}}^{2}=-\kappa$. In particular, considering the particular situation κ<0, it follows that for the warping function defined by $F(t)=t\sqrt{-\kappa }$, *m* is a $W_{2}$-curvature flat manifold and obviously the fiber $\overset{˜}{N}$ is an Einstein manifold.

Example 3 Example of perfect fluid space time: Quark-gluon is the closest known substance to a perfect fluid: perfect fluids are used in general relativity to model idealized distribution of matter, such as the interior of a star or an isotropic universe. In the latter case, the equation of state of the perfect fluid may be used in FLRW equation to describe the evolution of the universe.

Example 4 Example of shear-free, vorticity-free, and acceleration-free vector field: In a space-time of dimension ≻3 let $u_{k}$ be a smooth vector field that is shear-free, vorticity- free, and acceleration-free. Then $u_{k}u^{k}=-1$,

$$\nabla_{i}u_{j}=\psi \left(g_{ij}+u_{i}u_{j}\right),$$

*ψ* is a scalar function [34]. In other word, $u_{k}$ is a unit torse-forming vector field. Thus a torse-forming vector field is a smooth vector field which is shear-free, vorticity-free, and acceleration-free.

Example 5 Example of dark matter: Galaxy clusters can contain hundreds or thousands of galaxies, each of which have their own dark matter hola. One particular galaxy eluster, known as the Bullet cluster, provides some of the best evidence we have for the existence of dark matter. Using fluctuations in the cosmic microwave background (CMB) astronomers determined that dark matter is about 27% of the contents of the universe, in terms of its overall contributions to the total mass and energy content of the cosmos.

Example 6 Example of static space-time: Schwarzschild space-time is an example of a static space-time, since Schwarzschild solution is the only solution for any spherical symmetric vacuum field equations.

## 7 Conclusions

An obvious and useful extension of Cartesian product manifolds are WPMs. It should be remembered that Lorentzian WPMs are a common representation of many different kinds of space-times in general relativity. Singly WPMs include generalized Robertson-Walker space-times and conventional static space-times [36]. Hence, WPMs are important in general relativity as well as differential geometry. Warped product structure have therefore been extensively researched and expanded. As a result, studying the $W_{2}$-tensor on WPMs contributes a deeper comprehension of the WPMs.

This article has focused on studying the $W_{2}$ flatness and symmetry in the framework of WPMs. Here, we establish that the fiber manifold of a $W_{2}$ curvature flat WPM is an Einstein manifold and the fiber manifold of a $W_{2}$ symmetric WPM is a Ricci symmetric manifold. Also, in this study, we have clearly explained the physical importance of space-times using $W_{2}$-curvature flat GRW space-time.

In the future, we or perhaps other researchers will look at the characteristics of various curvature tensors on WPMs in relation to cosmological models, general relativity theory and modified gravity theory. Also, it would be valuable to explore the application of the findings from this research to scenarios involving multiply warped products. It is important to note that multiply warped products serve as a broader extension of warped products, enabling the creation of more diverse space-time models [35], [37].

## CRediT authorship contribution statement

**Abdallah Abdelhameed Syied:** Investigation, Funding acquisition, Formal analysis, Data curation, Conceptualization. **Uday Chand De:** Resources, Project administration, Methodology, Investigation. **Nasser Bin Turki:** Validation, Supervision, Software, Resources. **Gabriel-Eduard Vîlcu:** Writing – review & editing, Writing – original draft, Visualization, Validation, Supervision.

## Declaration of Competing Interest

The authors declare that they have no known competing financial interests or personal relationships that could have appeared to influence the work reported in this paper.

## Data Availability

No data was used for the research described in the article.
